# Wavelet-Based Bracketing, Time–Frequency Beta Burst Detection: New Insights in Parkinson’s Disease

**DOI:** 10.1007/s13311-023-01447-4

**Published:** 2023-10-11

**Authors:** Tanmoy Sil, Ibrahem Hanafi, Hazem Eldebakey, Chiara Palmisano, Jens Volkmann, Muthuraman Muthuraman, Martin M. Reich, Robert Peach

**Affiliations:** 1 Department of Neurology, University Hospital Würzburg (UKW), Josef-Schneider-Str. 11, 97080 Würzburg, Germany; 2 Department of Brain Sciences, Imperial College London, London, UK

**Keywords:** Parkinson’s disease, Beta oscillations, Low beta, High beta, Closed-loop control

## Abstract

Studies have shown that beta band activity is not tonically elevated but comprises exaggerated phasic bursts of varying durations and magnitudes, for Parkinson’s disease (PD) patients. Current methods for detecting beta bursts target a single frequency peak in beta band, potentially ignoring bursts in the wider beta band. In this study, we propose a new robust framework for beta burst identification across wide frequency ranges. Chronic local field potential at-rest recordings were obtained from seven PD patients implanted with Medtronic SenSight™ deep brain stimulation (DBS) electrodes. The proposed method uses wavelet decomposition to compute the time–frequency spectrum and identifies bursts spanning multiple frequency bins by thresholding, offering an additional burst measure, ∆f, that captures the width of a burst in the frequency domain. Analysis included calculating burst duration, magnitude, and ∆f and evaluating the distribution and likelihood of bursts between the low beta (13–20 Hz) and high beta (21–35 Hz). Finally, the results of the analysis were correlated to motor impairment (MDS-UPDRS III) med off scores. We found that low beta bursts with longer durations and larger width in the frequency domain (∆f) were positively correlated, while high beta bursts with longer durations and larger ∆f were negatively correlated with motor impairment. The proposed method, finding clear differences between bursting behavior in high and low beta bands, has clearly demonstrated the importance of considering wide frequency bands for beta burst behavior with implications for closed-loop DBS paradigms.

**Supplementary Information:**

The online version contains supplementary material available at 10.1007/s13311-023-01447-4.

## Introduction

Extracellular transmembrane current measures the electrical activity driven by cellular activity and can thus provide insights into the local neuronal population and processing. One of the major sources of transmembrane currents is the collective synaptic activity in the neurons from the vicinity of the electrode [1–3]. The simultaneous and instantaneous firing of cortical neurons superimposed at the same location give rise to potentials, also known as local field potentials (LFP), which can be recorded using micro and macro electrodes during deep brain stimulation surgeries [4–8] and due to recent developments, can also be chronically recorded by implantable pulse generators (IPG) [9, 10]. LFP recordings from basal ganglia nuclei have provided considerable insights about nodes of the motor circuits in patients with movement disorders such as Parkinson’s disease (PD). For example, the direct correlation between excessive neural synchrony in the beta band range (13–35 Hz) and motor impairment has been repeatedly observed in patients suffering from PD [11–16]. The attenuation in beta band power through dopaminergic medications or with deep brain stimulation therapy is associated with motor improvement [11, 17–24].

These findings encouraged several groups to leverage the exaggerated synchronization in the beta band as a biomarker to tailor stimulation parameters in a closed-loop delivery method [25–28] when power in beta exceeds a certain threshold; stimulation is ramped up progressively and then ramped down as soon as the power in the beta band returns below the threshold [26, 28, 29]. However, recently, there has been a shift in the way beta band has been analyzed [30, 31]. Currently, beta band activity is not believed to be tonically elevated but comprise phasic bursts of varying durations and magnitudes. These bursts are exaggerated in magnitude and duration for patients with PD [30, 32]. Studies have further shown that dopaminergic therapies, such as levodopa, decrease burst duration and burst probability [30, 32]. Critically, these studies target the highest power frequency bin in the beta band for a given patient. While these studies have been successful in measuring disease severity, the effects of treatments, and adapting stimulation, they do not capture possible heterogeneity in frequency among the bursts and inherently ignore potentially useful information present in wider beta band activity.

In this study, we outline a new robust framework for beta burst identification and analysis of LFP recorded from PD patients using chronically implanted IPGs. Our proposed framework considers full band characterization instead of a single frequency bin or a beta peak frequency as outlined in previous beta burst localization methods. Using data from 7 patients implanted with Medtronic SenSight™ DBS, we corroborate that the low beta band is pathological [33, 34]. We provide novel results for high beta band which negatively correlates with clinical severity and perchance does not contribute to the hallmark symptoms of PD.

## Methods

### Subjects

In this proof-of-concept study, seven subjects (1 female) with advanced PD were investigated. The mean age at the time of surgery was 57.57 ± 1.13 years, and the average MDS-UPDRS III score from Levodopa challenge test [35, 36] med off condition was 36.28 ± 1.09, and med on condition was 13.28 ± 0.75. Levodopa equivalent daily dose (LEDD) Pre-OP was 1296.2 ± 213.622, and one-year Post-OP was 396.6 ± 92.425 (Table 1). Data presented as mean ± SEM. Patients underwent microelectrode recording (MER)–aided DBS implantation surgery. Microelectrodes were used to localize the subthalamic nucleus (STN) borders for lead placement. The lead chosen was Medtronic Sensight™ B33005, implanted bilaterally in the STN and connected to Percept PC IPG. Medtronic Sensight™ is a new segmented lead from Medtronic which has the capability of bipolar LPF recording. All recordings were performed at minimum 3 months after implantation. Antiparkinsonian medications were withdrawn at minimum 12 h before the recordings, and the stimulation was paused at least 30 min before the recordings began. The Institutional Review Board of the University Hospital of Wuerzburg (103/20) approved the study, and we received informed consent from all patients involved in the study according to the Declaration of Helsinki [37].

**Table 1:** Patient demographics and clinical scores for subjects under investigation

**Patient ID**	**Sex**	**Disease duration (years)**	**Age**	**MDS UPDRS III med off**	**MDS UPDRS III med on**	**H&Y scale (pre-op)**	**LEDD (pre-op, post-op at 1 year)**
1	M	9	59	30	8	2	1200, 325
2	M	13	63	47	22	2	1811.5, 805
3	M	6	52	27	6	2	1067, 420
4	M	16	44	32	13	2	860, 80
5	M	17	68	35	15	4	2050, 452.5
6	F	7	62	46	16	2	1635, 405
7	M	7	55	37	13	2	450, 100

*UPDRS* Unified Parkinson’s Disease Rating Scale, *H&Y* Hoehn and Yahr Scale, *LEDD* levodopa equivalent daily dose

### Data Acquisition

Recordings were collected in the resting state with patients seated with eyes open, except for subject 3 who was lying in a bed.

LFPs were recorded in stimulation off state with the Brainsense Survey ring modality, which can record six bipolar channels per hemisphere in two passes one after the other, i.e., both stim compatible pairs 0–3, 1–3, and 0–2 for the right and 8–11, 9–11, and 8–10 for the left hemisphere and immediately adjacent pairs 0–1, 1–2, and 2–3 for the right and 8–9, 9–10, and 10–11 for the left hemisphere. Here, 0 and 8 are the lowermost contacts, whereas 3 and 11 are the uppermost contacts for the left and right hemispheres, respectively.

The sampling rate was 250 Hz, and LFPs were recorded for 20.9 s which is the longest duration of sensing available in Brainsense Survey ring modality. All available contacts were used in all our analysis. For analysis of longer duration recordings in indefinite streaming modality, a supplementary figure (Supplementary 3) is provided.

### Signal Preprocessing

The recorded time-domain signals were analyzed offline using MATLAB scripts (v2022b, MathWorks) and the FieldTrip toolbox [38]. All time domain signals were high pass filtered at 1 Hz to remove low-frequency baseline drift from the signals.

**Fig. 1:**
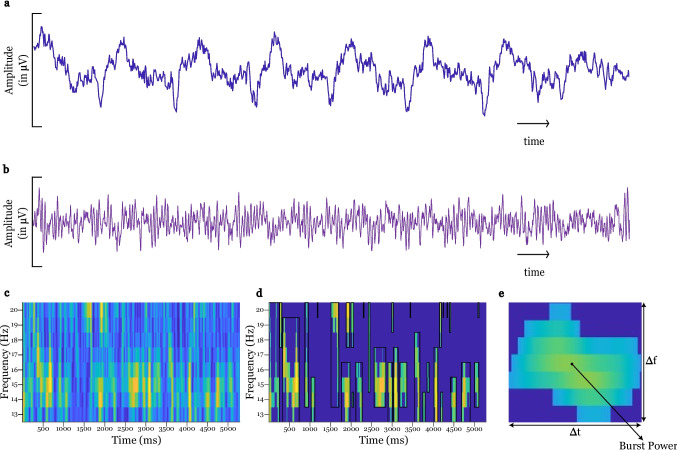
Workflow for identifying wider beta-band bursts in an example recording from a single channel. **a** Example recording signal containing ECG artifacts. **b** Signal after preprocessing, including high pass filtering and ECG removal. **c** Wavelet transformation yielding time–frequency decomposition. **d** Thresholding the signal at the 80th percentile yields individual bursts that span both time and frequency. Dark blue areas symbolize areas below the threshold. **e** Measurable quantities of the bursts. Burst duration (∆t): width (in milliseconds) of the bounded region. $\Delta$frequency (∆f): the height of the bounded regions (in Hz). Burst power: maximum magnitude (in a.u.) of a burst

LFP recordings from subjects 2, 5, and 6 were contaminated with ECG artifacts (example in Fig. 1a). These artifacts were removed using template identification using matched filtering and then performing a singular value decomposition on the identified template [39–41]. For a given hemisphere and channel(s), the time domain data is low passed at 15 Hz. The first few seconds of the low passed signal are used for visualization and localization of the beginning of QRS complexes of the ECG-contaminated signal. Once the beginning of the QRS complex is localized, we create epochs of 560 ms (based on 460 ms from the upper limit of QT interval and an added buffer of 100 ms [41]) from the original signal. These epochs are then used as delayed input templates in a matched filter configuration. The templates are flipped to obtain the FIR filter coefficients, and the entire time domain signal is filtered using the previously obtained coefficients. Then, from the resulting filtered signal output, the peaks (the peaks denote the greatest correlation between the input template and the original signal) and the location of the peaks are located with the constraint that the peaks are at least 0.5 s apart, i.e., 120 beats/second. For all the matched filter outputs, the output with the largest cumulative average peak is selected, and ECG-corrupted LFP templates are extracted from the original signal based on the location of peaks located. Since the peaks denote the beginning of QRS, we obtain the ECG-corrupted LFP templates from the original signals between time points − 0.2 s (for P wave) and + 0.46 s (for QRS) with the 0^th^ point being the beginning of QRS.

Once the ECG-corrupted LFP templates are extracted, they are fed to the singular value decomposition (SVD) algorithm which decomposes them into eigenvectors that are subsequently visually inspected. Eigenvectors that closely resemble the QRS wave in the ECG are noted and are used to reconstruct artifacts. The reconstructed artifact is then subtracted from each ECG corrupted LFP template and is then copied back to their timestamps in the original signal. Usually, only the largest eigenvector and, in rare cases, the two largest eigenvectors are used to reconstruct the artifacts.

The time points of the ECG-corrupted LFP templates are also used for the respective contralateral hemisphere (if ECGs are visually found) to extract ECG-corrupted LFP templates from the contralateral hemisphere which are then used for singular value decomposition and subsequent inspection, reconstruction, and subtraction to obtain the ECG-free LFP template which can be replaced in the original time series (example in Fig. 1b).

### Time–Frequency Decomposition

To calculate the time–frequency spectrum of a time domain signal, we applied a wavelet transform using the MATLAB Wavelet toolbox with Morlet wavelets (width = 10) between 10 and 40 Hz and a frequency resolution of 1 Hz. A width of 10 cycles was chosen as an optimal tradeoff between time and frequency resolution. The power of each frequency bin was smoothed using the Savitzky-Golay filter with span of 0.2 s.

### Time–Frequency Burst Characterization

Our framework to characterize beta bursts generalizes the method developed by Tinkhauser et al. [32], where they use a single frequency bin and define beta bursts as regions in time that exceed a given amplitude threshold. Here, we instead define bursts as regions in the time–frequency spectrum that exceed a given 2-dimensional threshold.

The time–frequency spectrum is a matrix describing the power at a given frequency (*y*-axis) and time (*x*-axis) (see Fig. 1c). The time–frequency matrix was subsequently binarized using a power threshold to create a mask that describes regions of the spectrum that deviate in power, which we consider as beta bursts (Fig. 1d).

The threshold chosen for binarization was the 80th percentile of the power in each channel, meaning that 80 percent of the data below the threshold will be discarded and we only consider the remaining 20 percent of the data above the threshold. The threshold was kept at the 80th percentile for all patients and across all channels. The choice of threshold is relatively arbitrary; however, Tinkhauser et al. [32] show that the choice of threshold did not greatly affect results.

The binarized mask is then used to filter the time–frequency spectrum, retaining only those regions that exceeded the chosen power threshold. Bursts straddle multiple frequency bins and stretch across time (Fig. 1c). To define a burst, we search for connected components in the binarized mask matrix (function *bwconncomp* MATLAB). Non-zero elements are connected if they are incident directly or diagonally. Finally, for each burst, we identify the bounding region (function *regionprops*, MATLAB).

Since beta band has been segregated into two different bands in previous studies, to characterize the behavior of beta bursts, we have used different sub-ranges within the beta band range, including the low beta band (13–20 Hz) and the high beta band (21–35 Hz) [42] as sub-ranges within the beta band range.

### Metrics

For each burst and its bounding region, we calculate three different metrics:

- Burst duration (∆t): width (in milliseconds) of the bounded region. Indicates the duration of the burst, i.e., how long that burst was active
- $\Delta$frequency (∆f): the height of the bounded regions (in Hz), indicating the maximum range of frequencies that were active for a given burst
- Burst power: maximum magnitude (in a.u.) of a burst. Scaled and measured in arbitrary units

### Statistical Analysis

All statistical analyses were done using MATLAB v2022b and JASP v0.17.1. All data presented as mean ± standard error of the mean (SEM) unless otherwise stated.

#### Across Burst Metrics

Δt vs burst power, ∆f vs Δt, and ∆f vs burst power were correlated for each bipolar contact pair and patient separately for each hemisphere, using Fisher z-transformed Pearson correlation coefficient which measures linear relationship strength between two variables. *P* values for multiple comparisons were corrected using Benjamini-Hochberg (1995) procedure using a false discovery rate of 0.05.

#### Between Burst Metric and Clinical Score

To compute clinical correlation of ratio of Δt or Δf in different intervals with MDS UPDRS III med off scores, we have used Fisher *z*-corrected Pearson correlation coefficient. We consider all available contact pairs within both hemispheres for all patients and find the average Fisher *z*-corrected correlation coefficient across hemispheres.

Formulation of the ratio of bursts is as follows:

$$\text{The ratio of } \Delta \text{t}=\frac{\text{No}. \text{ bursts} \text{ with} \text{ duration} \text{ that} \text{ falls} \text{ within } \text{a } \text{time } \text{interval}}{\text{Total } \text{number } \text{of } \text{bursts}}$$

$$\text{The } \text{ratio } \text{of } \Delta \text{f}=\frac{\text{No}. \text{ bursts } \text{with } \Delta \text{f } \text{that } \text{falls } \text{within } \text{a } \text{frequency } \text{interval }}{\text{Total } \text{number } \text{of } \text{bursts}}$$

For example, we have 20 bursts whose duration lies in the interval 0.1–0.2 s. The total number of bursts of any duration for that contact and hemisphere is 100. The ratio of Δt in this interval will be 20/100 = 0.2.

#### For Distribution of Burst Metrics

To compare the distributions of bursting for different Δt windows between conditions (i.e., between low and high beta), we performed a two-way repeated measures ANOVA with a 10 × 2 design, i.e., ten time windows and two conditions, and for different Δf windows between conditions, we performed a two-way repeated measures ANOVA with a 4 × 2 design, i.e., four Δf windows and two conditions. Normality checks were done using Shapiro–Wilk test where significant results suggest deviation in normality. For the post hoc test of burst properties, we have used the Mann–Whitney *U*-test since some intervals showed significant results for deviation in normality.

#### For All Other Comparisons

For comparing burst probability between bands, we have used a two-sample *t*-test. For correlation between Sorensen-Dice similarity (Sorensen-Dice index looks for similarity or diversity between two binarized sets by dividing twice the area of intersection between the sets and the sum of areas of the sets) scores between bands across hemispheres, we have used Pearson correlation.

## Results

### Δt, Burst Power, and Δf Are Strongly Correlated with Each Other 

**Fig. 2:**
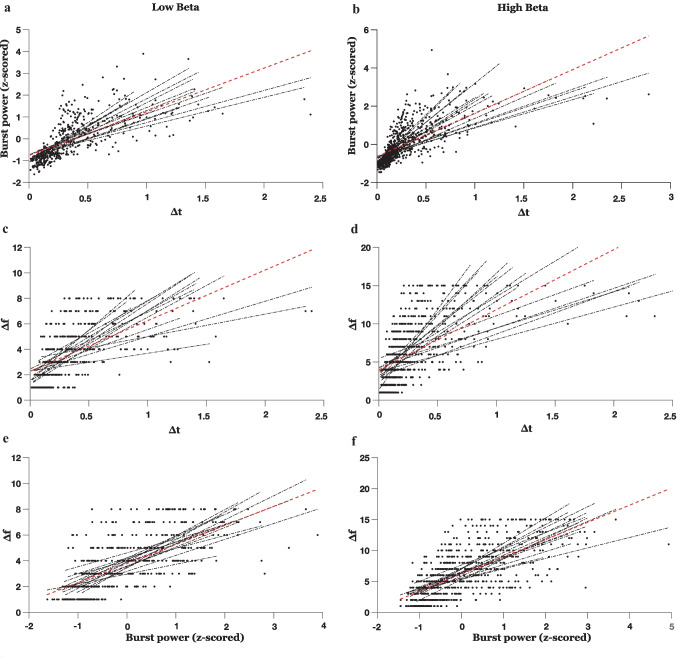
Correlation between burst metrics for low (**a**,** c**,** e**) and high (**b**,** d**,** f**) beta bands separately. Strong correlations are observed across all metrics: Δt, burst power, and ∆f. Dashed black lines indicate a linear fit to each hemisphere of each patient individually. For simplicity, we only show the electrodes with the highest beta band power in their respective bands and hemispheres. Dashed red lines indicate a linear fit to all bursts across all hemispheres and patients together. Δt is strongly correlated with burst power (mean Fisher-transformed *r* value = 0.97 ± 0.059, all *p* values smaller than FDR corrected critical *p* = 0.011, in low beta band; mean Fisher-transformed *r* value = 1.11 ± 0.057, all *p* values smaller than FDR corrected critical *p* = 9.048e-06, in high beta band) and with $\Delta$f (mean Fisher-transformed *r* value = 0.88 ± 0.054, all *p* values smaller than FDR corrected critical *p* = 0.009, in low beta band; mean Fisher-transformed *r* value = 0.88 ± 0.04, all *p* values smaller than FDR corrected critical *p* = 0.003 in high beta band). Burst power is strongly correlated with $\Delta$f (mean Fisher-transformed *r* value = 0.91 ± 0.072, all *p* values smaller than FDR corrected critical *p* = 0.016, in low beta band and mean Fisher-transformed *r* value = 0.93 ± 0.064, all *p* values smaller than FDR corrected critical *p* = 9.77e-04, in high beta band)

Figure 2 shows the relation between different burst characteristics in both low and high beta bands. Δt is strongly correlated with burst power (mean Fisher-transformed *r* value = 0.97 ± 0.059, all *p* values smaller than FDR corrected critical *p* = 0.011, in low beta band; mean Fisher-transformed *r* value = 1.11 ± 0.057, all *p* values smaller than FDR corrected critical *p* = 9.048e-06, in high beta band) and with $\Delta$f (mean Fisher-transformed *r* value = 0.88 ± 0.054, all *p* values smaller than FDR corrected critical *p* = 0.009, in low beta band; mean Fisher-transformed *r* value = 0.88 ± 0.04, all *p* values smaller than FDR corrected critical *p* = 0.003 in high beta band).

Burst power is strongly correlated with $\Delta$f (mean Fisher-transformed *r* value = 0.91 ± 0.072, all *p* values smaller than FDR corrected critical *p* = 0.016, in low beta band; mean Fisher-transformed *r* value = 0.93 ± 0.064, all *p* values smaller than FDR corrected critical *p* = 9.77e-04, in high beta band).

### Clinical Correlations Are Reversed for High and Low Beta Band

Clinical correlations were performed between (i) the ratio of Δt and (ii) the ratio of $\Delta$f vs the MDS UPDRS III scores in high and low beta bands to examine how bursting in each band relates to the severity of disease and motor scores.

**Fig. 3:**
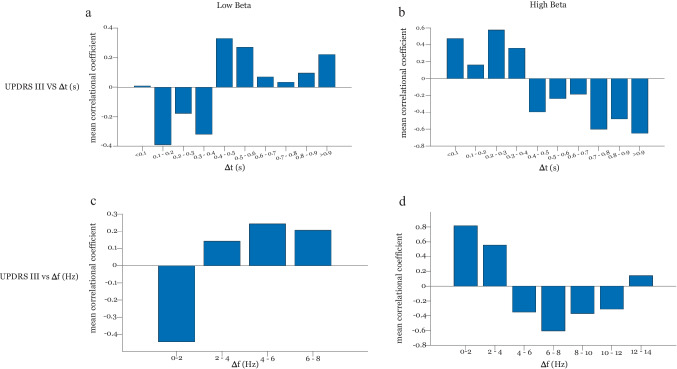
Fisher transformed Pearson’s correlation *r* values between MDS UPDRS III med off scores and Δt and $\Delta$f windows in low beta band (**a** and **c** respectively) and high beta band (**b** and **d** respectively)

In low beta band (13–20 Hz), longer Δt is positively correlated with motor scores (Fig. 3a), while in high beta band (21–35 Hz), longer Δt correlates negatively with motor scores (Fig. 3b).

The same relation is true for the correlation between MDS UPDRS III and $\Delta$f, where $\mathrm{larger }\Delta$f and UPDRS III are positively correlated in the lower beta band (Fig. 3c) and negatively correlated in the higher beta band (Fig. 3d).

A separate supplementary Fig. (3) is provided for clinical correlations between (i) the ratio of Δt and (ii) the ratio of $\Delta$f vs the MDS UPDRS III scores in high and low beta bands for indefinite streaming mode of the IPG which allowed us to record for longer durations (> 1 min and in some cases > 2 min). Using indefinite streaming restricts, the number of available contacts to record signals from six (6) to three (3) (i.e., 0–3, 1–3, and 0–2 and 8–11, 9–11, and 8–10 for right and left hemispheres respectively).

### Significance of Burst Features in Low and High Beta Band

**Fig. 4:**
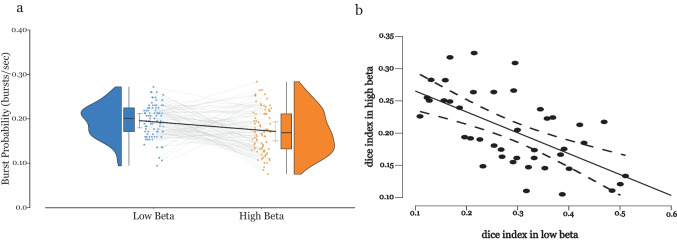
**a** Burst probability in low and high beta bands normalized by the width of the frequency band. In low beta band, bursts have a significantly higher likelihood (*mean* = 0.2 ± 0.004) of occurring than bursts in the high beta band (*mean* = 0.17 ± 0.005), with *p* < 0.001. **b** Dice index between hemispheres in low and high beta band. In low beta band: low left vs low right hemisphere. In high beta band: high left vs high right hemisphere. Bursts in the low beta (*mean* = 0.291 ± 0.017) look more similar than bursts in high beta band (*mean* = 0.203 ± 0.009) across hemispheres with Pearson’s *r* − 0.606 and *p* < 0.001

We calculated the burst probability in low and high beta bands (normalized by the width of the band, i.e., the maximum possible $\Delta$f of the band, 8 Hz for low beta and 15 Hz for high beta) as shown in Fig. 4a. In low beta band, bursts have a significantly higher likelihood (*mean* = 0.2 ± 0.004) of occurring than bursts in the high beta band (*mean* = 0.17 ± 0.005), with *p* < 0.001.

We looked for the similarity between both hemispheres with regard to beta bursts, i.e., if bursts are bilaterally synchronized between hemispheres in both bands, using the Sorensen-Dice index (Fig. 4b). We compute the dice index for all electrodes in both hemispheres for a patient between both bands, i.e., low left vs low right and high left vs high right for each patient. We then use Pearson’s correlation to find if there is a correlation between similarity of bursts in low and high beta bands and found that bursts in the low beta (*mean* = 0.291 ± 0.017) look more similar than bursts in high beta band (*mean* = 0.203 ± 0.009) across hemispheres with Pearson’s *r* − 0.606 and *p* < 0.001.

**Fig. 5:**
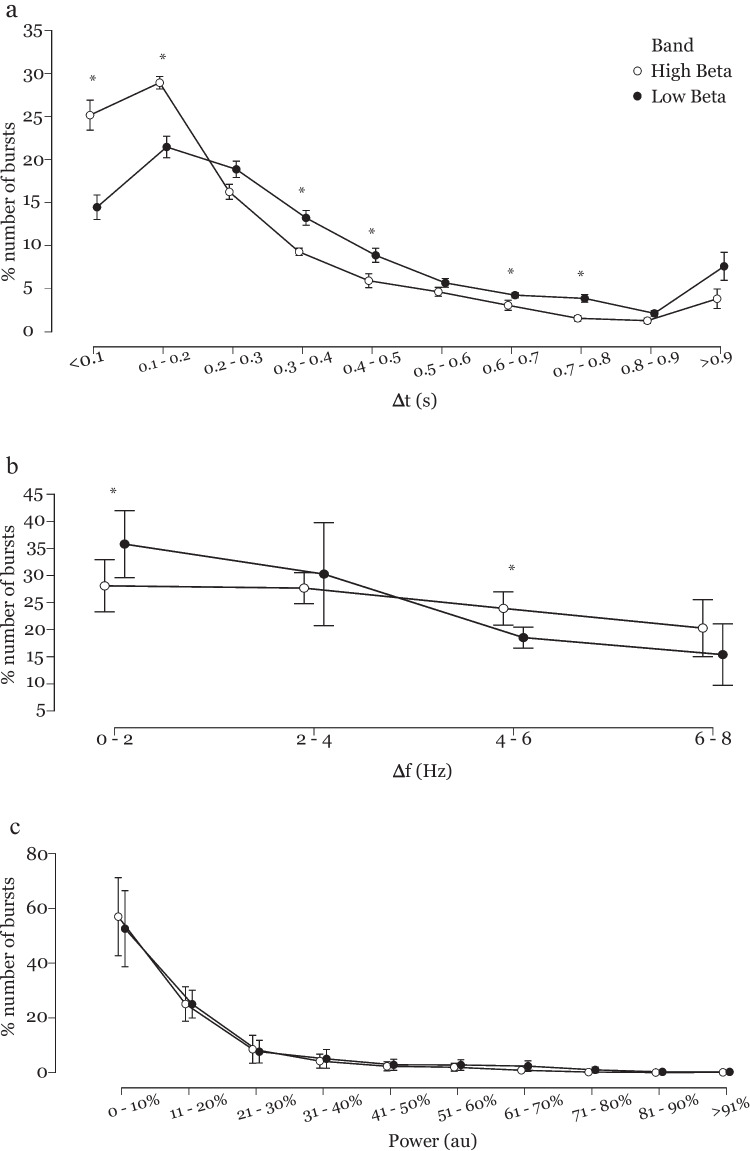
Distribution of burst properties for high and low beta bands. **a** Percentage of bursts by time window. **b** Percentage of bursts in each $\Delta$f window. **c** Percentage of bursts by power (windows increase in 10% increments, divided into equal power bins, 100% refers to the maximum power burst across all the bursts) *p < 0.05. Post hoc test (Mann–Whitney) of Δt between low and high beta shows that percentage amount of bursts in duration windows < 0.1 s [U(12) = 49, *p* < 0.001], 0.1–0.2 s [U(12) = 49, *p* = 0.002] are significantly higher in high beta and amount of bursts in duration windows 0.2–0.3 s [U(12) = 7, *p* = 0.026], 0.3–0.4 s [U(12) = 1, *p* = 0.001], 0.4–0.5 s [U(12) = 8, *p* = 0.04], 0.6–0.7 s [U(12) = 8, *p* = 0.04], and 0.7–0.8 s [U(12) = 1, *p* = 0.001] are significantly higher in low beta. Post hoc test of $\Delta$f windows between low and high beta shows the percentage amount of $\Delta$f windows 0–2 Hz [U(12) = 5, *p* < 0.01] to be significantly higher in low beta and 4–6 Hz [U(12) = 47, *p* = 0.002] to be significantly higher in high beta. Power of the bursts do not show significant differences between bands

Repeated measure ANOVA (RM-ANOVA) shows significant main effect interaction between condition (low and high beta) and Δt [F (df = 2.3) = 15.089, *p* < 0.001] and between condition (low and high beta) and $\Delta$f [F (df = 1.794) = 4.072, *p* = 0.03]. RM-ANOVA for percentage number of bursts divided by power bins does not show a significant difference between conditions. A post hoc test (Mann–Whitney) of Δt between low and high beta shows that percentage amount of bursts in duration windows < 0.1 s [U(12) = 49, *p* < 0.001] and 0.1–0.2s [U(12) = 49, *p* = 0.002] are significantly higher in high beta, and amount of bursts in duration windows 0.2–0.3 s [U(12) = 7, *p* = 0.026], 0.3–0.4 s [U(12) = 1, *p* = 0.001], 0.4–0.5 s [U(12) = 8, *p* = 0.04], 0.6–0.7 s [U(12) = 8, *p* = 0.04], and 0.7–0.8 s [U(12) = 1, *p* = 0.001] are significantly higher in low beta. Post hoc test of $\Delta$f windows between low and high beta shows the percentage amount of $\Delta$f windows 0–2 Hz [U(12) = 5, *p* < 0.01] to be significantly higher in low beta and 4–6 Hz [U(12) = 47, *p* = 0.002] to be significantly higher in high beta (Fig. 5).

## Discussion

This study presents a novel data-driven method of identifying, visualizing, measuring, and classifying beta bursts in chronic electrophysiological recordings from PD patients. We use this method to compare burst behavior in low and high beta bands and reveal their contrasting correlation with motor impairment scores. We further discuss the potential pathological and physiological nature of high and low beta bands.

### Wavelet-Based Bracketing, Time–Frequency Beta Burst Detection

Existing tools for beta burst detection are restricted to a single frequency bin, limiting their ability to study patients with distributed beta peaks [31, 32]. Here, we introduce a data-driven method that detects bursts for a wide frequency band, inclusive of patients with single and distributed beta peaks. By studying beta bursts in both the time and frequency domains, our method naturally offers an additional beta burst measure, namely, ∆f, that captures the width of a burst in the frequency domain. Our method can be viewed as a generalization of existing methods, and thus, where appropriate, our results are consistent with previously described beta detection techniques [31, 32].

### Bursts in Low and High Beta Behave Differently

Classically beta band has been segregated between low (13–20 Hz) and high beta band (21–35 Hz) [42]. Low beta has been shown to be modulated by both antiparkinsonian medications and electrical stimulation using DBS [13, 23, 42, 43], while high beta band is not attenuated by antiparkinsonian medications and electrical stimulation and is speculated to act as a preferential link between STN and cortex [44–46]. In our study, we found bursts with longer duration and larger ∆f in low beta (13–20 Hz) from the electrophysiology recordings to be positively correlated with UPDRS III scores, while bursts with longer durations and larger ∆f in high beta (21–35 Hz) are negatively correlated with UPDRS III scores. Tinkhauser et al. found that bursts of longer duration are positively correlated with UPDRS, which reflects our findings in low beta but contrast with our results in high beta suggesting that low beta could be pathological [32].

### Distribution and Likelihood of Bursts Between Low and High Beta

In addition to burst duration Δt and burst magnitude, we can also use $\Delta$f as a placeholder for synchrony [32, 47]. We speculate that smaller $\Delta$f is a result of local synchrony (greater for low beta), whereas larger $\Delta$f showcases wider synchrony (greater for high beta) in the nuclei. Bursts in low beta also have significantly greater likelihood of occurrence than in high beta. The greater likelihood of occurrence of bursts in low beta likely increases synchrony, duration, and power in a cluster, conceivably reinforcing each other, an observation that has been made in other studies [33]. We find that burst power is strongly correlated to the duration of the burst, yet the power of the bursts do not show significant differences between bands and therefore cannot be reliably used as a discriminant as evident in previous studies [30, 48]. Studies have also shown greater bilateral synchronization between hemispheres in subjects with PD in the beta peak frequency [32, 49], but we report that the coherence is a result of greater synchronization in low beta.

### Low Beta Can Be Considered the Culprit

As explained by Brittain and Brown, Little and Brown, and Hanslmayr et al., excessive synchrony in a system leads to two outcomes [47, 50, 51]. First, an increase in excitatory post-synaptic potentials leads to an increase in temporal/spatial summation in neurons and thus increases the chances of firing in downstream neurons. Efficient firing comes at the expense of decreased entropy, which in a spatially isolated system can lead to efficient information flow among parallel channels. Second, an increase in synchrony in a spatially integrated system leads to decreased entropy and thus decreased information flow which in turn reduces the parallel processing capability of the system. As a result of long-held speculations of the presence of multiple functionally segregated bands within the basal ganglia–cortical loop which are responsible for different functions [52, 53], we hypothesize that high beta band is a result of the first outcome of synchrony, i.e., increase of synchrony in a spatially isolated system, and low beta band a result of the second outcome of synchrony, i.e., increase of synchrony in a spatially integrated system.

A decrease in high beta power as observed during movement, high coherence between STN and motor cortex in high beta band, i.e., acts as a preferential link between STN and cortex, immutability to attenuation on intake of dopaminergic medications and stimulation, and negative correlation of longer bursts with clinical severity scores, reinforces our belief that high beta band is a maintainer of status quo as described by Engel and Fries, i.e., oscillations are stronger when maintenance of status quo is necessitated or anticipated [44–46, 53–55]. However, low beta is modulated by intake of antiparkinsonian medications and by stimulation, does not decrease during movement, has phase-amplitude coupled within the subthalamic nucleus, and is positively correlated with longer bursts and is a pathological band, i.e., oscillations in low beta limit the information coding capacity of STN [13, 23, 42, 53, 55–57]. Evidence also points towards a reduction in high beta on levodopa administration, but this is possibly due to the nonlinear modulation of high beta by low beta, and any suppression of high beta is a result in the decrease of the nonlinear correlation found between the bands [42, 58, 59]. The justification for low beta is still speculative as we do not know how information flow occurs in low beta and how a restrictive information coding scheme leads to the hallmark symptoms of PD. An alternative explanation for the inverse relation between high beta and MDS UPDRS III medication off scores could be that low beta reflects the degree of circuit derangement, whereas high beta is a compensatory mechanism. To find experimental evidence for the proposed roles of low and high beta, we would like to analyze information flow with simultaneous deep brain recordings and high-resolution EEG in the future.

Various studies have shown a shift from longer to shorter durations, larger to smaller mean magnitudes of bursts, and a decrease in bilateral symmetry following intake of dopaminergic medication [32, 60, 61]. We could also hypothesize a similar effect for $\Delta$f, i.e., a shift from longer to shorter $\Delta$f signifying a decrease in synchrony and possibly pointing a shift from pathological to physiological signaling. However, here, our recordings were restricted to medication off states and instead leave this to future studies.

## Limitations

While our method can detect bursts across wide frequency bands, by thresholding, we are implicitly limited to the peak power in a band, limiting information from power values below the threshold and thus restricting the signal-to-noise ratio. Another limitation we faced is the sample size and the unavailability of medication on electrophysiology recordings which we hope to include in future longitudinal studies.

## Conclusion

In summary, we present a novel data-driven method for broadband beta burst detection. Using the proposed method, we were able to separate the contributions of low and high beta band bursts and further able to introduce a novel burst metric $\Delta$f. We found that longer burst durations and larger $\Delta$f in low beta correlated positively with larger motor scores offering an alternative approach for optimizing DBS programming. By identifying contacts with the longest duration or largest $\Delta$f bursts, we can offer a potentially more robust alternative for open loop configuration than methods based on maximal beta peaks or fiber tract activation. Additionally, our wavelet-based approach has the potential to be used in a multi-input single-output closed-loop paradigm to optimize deep brain stimulation, where $\Delta$f offers an additional input in comparison to existing beta burst detection methods.

### Supplementary Information

Below is the link to the electronic supplementary material.

Supplementary file1 (DOCX 1355 KB)

## Data Availability

Patients’ data used for the study could not be shared because of the agreement signed with the participants. However, partially analyzed, deidentified electrophysiological data could be shared with appropriate request to corresponding author.
