# Supplementary material for: Wavelet-Based Bracketing, Time–Frequency Beta Burst Detection: New Insights in Parkinson’s Disease
Source: Neurotherapeutics. 2023 Oct 11;20(6):1767–78. doi: 10.1007/s13311-023-01447-4 (PMC10684463; doi:10.1007/s13311-023-01447-4)
Supplement: Supplementary file 1 — Supplementary file1 (DOCX 1355 KB) [file 13311_2023_1447_MOESM1_ESM.docx]

**
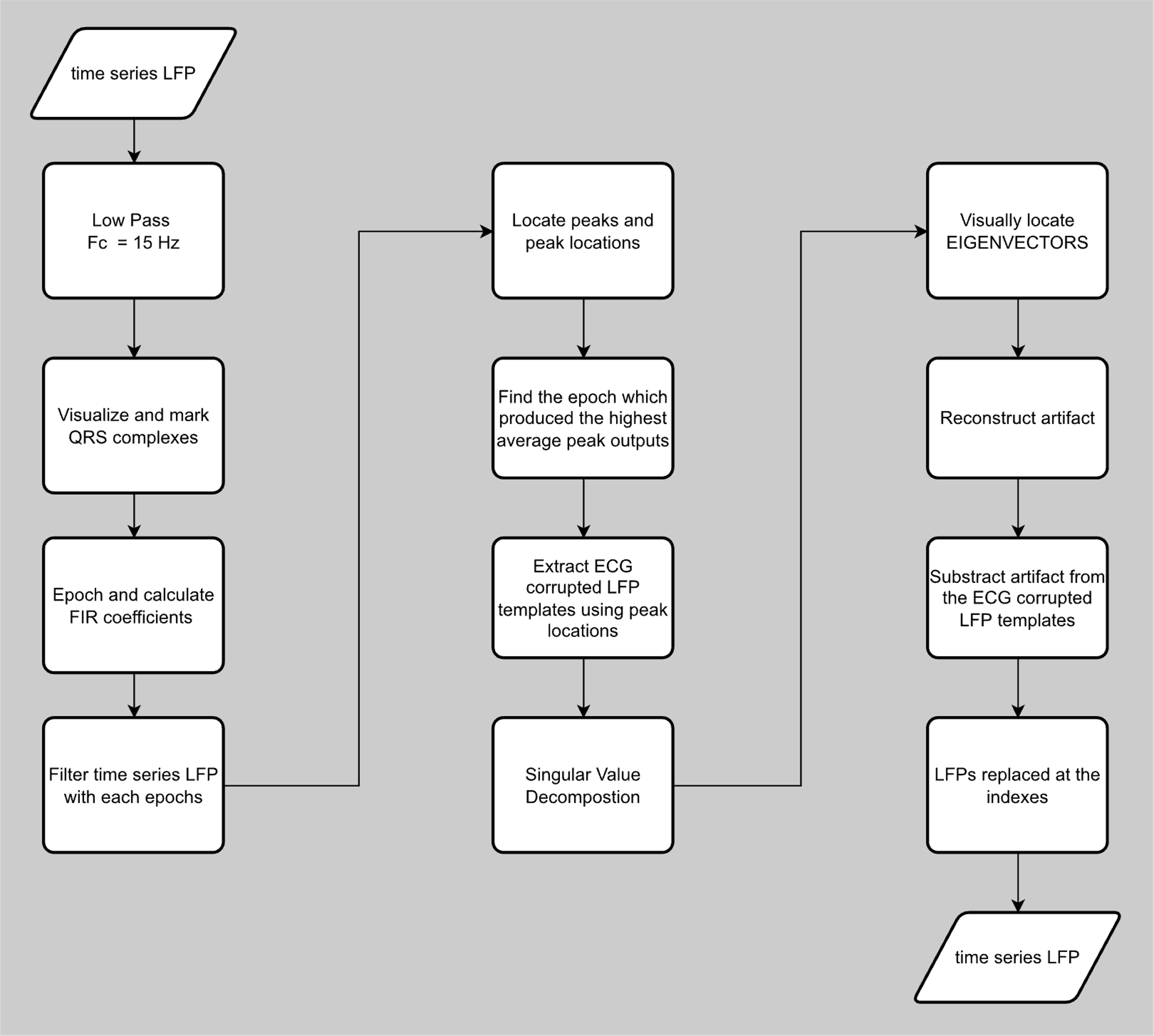
Supplementary Information**

**Supplementary 1**: ECG removal flowchart. Shows the flowchart for ECG detection, visualization, and removal in ECG corrupted LFP signals.


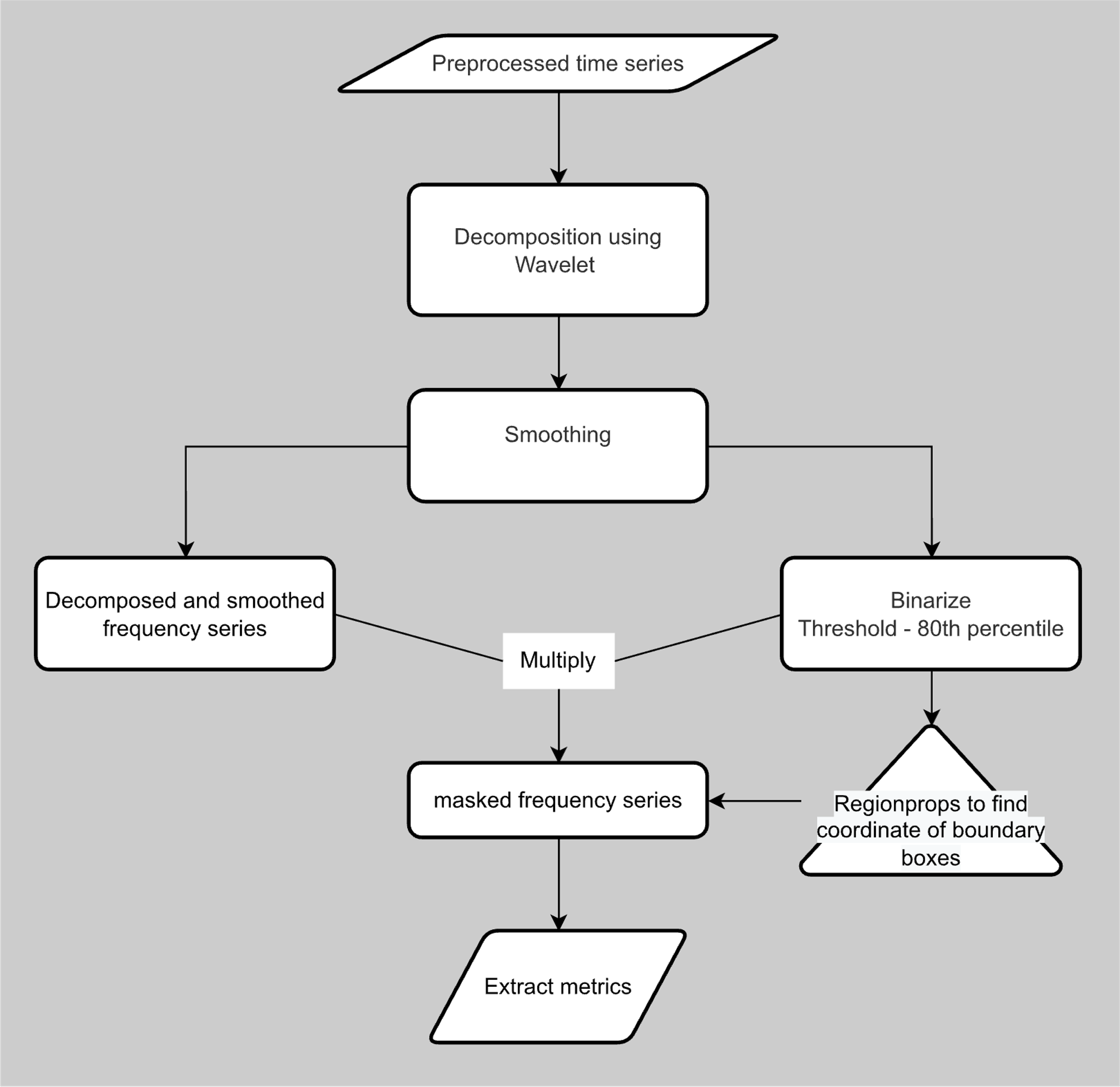
**Supplementary 2**: Burst detection flowchart. Shows the steps to detect beta burst in a preprocessed signal.

**Supplementary 3:** Fisher transformed Pearson’s correlation r values between MDS UPDRS III med off scores and Δt and $\Delta$f windows in low beta band (a and c respectively) and high beta band (b and d respectively) in indefinite streaming mode of the IPG which allowed us to record for longer durations (>1 minute, and in some cases >2 minutes) but the available contacts are reduced from six (6) to three (3) (i.e., 0-3, 1-3, 0-2 and 8-11, 9-11, 8-10 for right and left hemispheres respectively).
